# Design and study protocol of the maternal smoking cessation during pregnancy study, (M-SCOPE)

**DOI:** 10.1186/1471-2458-11-903

**Published:** 2011-12-06

**Authors:** Andriani N Loukopoulou, Constantine I Vardavas, George Farmakides, Christos Rossolymos, Charalambos Chrelias, Manolis N Tzatzarakis, Aristidis Tsatsakis, Maria Lymberi, Gregory N Connolly, Panagiotis K Behrakis

**Affiliations:** 1 Smoking and Lung Cancer Research Center, Hellenic Cancer Society, Athens, Greece; 2 School of Medicine, National and Kapodistrian University of Athens, Athens, Greece; 3 Center for Global Tobacco Control, Division of Society, Human Development and Health, Harvard School of Public Health, Boston, USA; 4 Peripheral General Maternity Hospital 'Elena Venizelou', Athens, Greece; 5 Maternity Unit, 'Attikon' University Hospital, Athens, Greece; 6 Laboratory of Toxicology, School of Medicine, University of Crete, Heraklion, Greece

## Abstract

**Background:**

Maternal smoking is the most significant cause of preventable complications during pregnancy, with smoking cessation during pregnancy shown to increase birth weight and reduce preterm birth among pregnant women who quit smoking. Taking into account the fact that the number of women who smoke in Greece has increased steadily throughout the previous decade and that the prevalence of smoking among Greek females is one of the highest in the world, smoking cessation should be a top priority among Greek health care professionals.

**Methods/Design:**

The Maternal Smoking Cessation during Pregnancy Study (M-SCOPE), is a Randomized Control Trial (RCT) that aims to test whether offering Greek pregnant smokers a high intensity intervention increases smoking cessation during the third trimester of pregnancy, when compared to a low intensity intervention. Prospective participants will be pregnant smokers of more than 5 cigarettes per week, recruited up to the second trimester of pregnancy. Urine samples for biomarker analysis of cotinine will be collected at three time points: at baseline, at around the 32^nd ^week of gestation and at six months post partum. The control group/low intensity intervention will include: brief advice for 5 minutes and a short leaflet, while the experimental group/intensive intervention will include: 30 minutes of individualized cognitive-behavioural intervention provided by a trained health professional and a self-help manual especially tailored for smoking cessation during pregnancy, while counselling will be based on the ''5 As.'' After childbirth, the infants' birth weight, gestational age and any other health related complications during pregnancy will be recorded. A six months post-partum a follow up will be performed in order to re-assess the quitters smoking status.

**Discussion:**

If offering pregnant smokers a high intensity intervention for smoking cessation increases the rate of smoking cessation in comparison to a usual care low intensity intervention in Greek pregnant smokers, such a scheme if beneficial could be implemented successfully within clinical practice in Greece.

**Trial Registration:**

ClinicalTrials.gov Identifier NCT01210118

## Background

### Tobacco use among women and during pregnancy

Tobacco consumption is the main cause of preventable death globally with tobacco considered to be the only consumer product legally on sale that kills such a high percentage of its users [1]. Epidemiological studies over the last 30 years have concluded that the total mortality of smokers is increased within the range of 50% to 116%, depending on the smokers' age, their average tobacco consumption and their years as a smoker [2]. As a result smoking kills one third to half of all smokers, who die on average 15 years earlier than their non smoking peers [3]. In 2000 it was estimated that 4.83 million premature deaths worldwide were caused by smoking, with the main causes of death attributable to: cardiovascular disease, lung cancer and chronic obstructive pulmonary disease, with the total number of deaths attributable to tobacco use greater than the number of deaths due to drug use, alcohol consumption, AIDS, car accidents, suicides and murders combined [4-6].

While, it has been estimated that 10 million female deaths were attributable to tobacco use between 1950 and 2000, the projected number of deaths attributable to smoking among women over the next 30 years is estimated to be more than double that of the previous 50 years [7]. This increase in mortality is attributable to the increase in female smoking rates, which subsequently leads to a higher percentage of women who smoke during their reproductive years and thus during the early stages of pregnancy (before it being verified) and a smaller percentage will continue to smoke throughout the gestational period.

Maternal smoking during pregnancy is also the most significant cause of preventable complications during pregnancy [8]. Active maternal smoking has been associated with a number of adverse pregnancy outcomes, such as premature birth and perinatal mortality [9]. The perinatal mortality rate has been identified to be 150% greater when the mother is a smoker [10], and it has been suggested that smoking is responsible for 15% of all cases of premature birth [11]. Moreover, perinatal mortality is increased among the offspring of pregnant smokers regardless of the number of cigarettes daily smoked, while this risk is reduced to an important degree when pregnant women stop smoking before the last trimester of pregnancy [12].

Since the first research performed in 1957[13], maternal active smoking during pregnancy has been shown to effect fetal birth weight and fetal growth (such as in height, head perimeter, perimeter of thorax and shoulders) and thus affect the growth of the lungs and brain, with possible ramifications that could continue into later life [14-17]. Active maternal smoking during pregnancy has been also shown to predispose infants towards the development of other health related issues, such as Sudden Infant Death Syndrome (SIDS) [18,19], infant's respiratory function [20-22] aid the development of asthma in childhood [23] and asthmatic bronchitis during the first year of life [24].

### Smoking cessation interventions during pregnancy and their benefits

Recognizing the importance of this problem, the issue of aiding or promoting smoking cessation during pregnancy is imminent. Furthermore, smoking cessation during pregnancy is economically beneficial, due both to the improvement of maternal health, as also due to its direct impact on improving infants' health [25]. Research has indicated that women who quit smoking during the first 3-4 months of pregnancy, give birth to infants of similar weight to those that never smoked [26,27]. As assessed through a recent systematic review carried out by Lumley et al. interventions for smoking cessation increase the mean birth weight of infants by 33 g (95% CI 11 g to 55 g) and simultaneously reduce preterm birth (pooled RR 0.84,95% CI: 0.72 to 0.98) [28]. These children during their development are likely to have a reduced need for health care and suffer less from chronic diseases and thus in general be of greater benefit to the health care system [29,30]. Indeed, if all pregnant women who smoke 15 or more cigarettes per day stopped smoking it has been estimated that 5% of hospital admissions of children less than 8 months could be prevented [31].

While 40% of pregnant women stop smoking spontaneously prior to their first visit to their gynaecologist and the verification of their pregnancy, the remaining continue to smoke, with the majority known to simply reduce their cigarette consumption [25,32-34]. It is these women that continue to smoke during pregnancy which should be targeted to participate in smoking cessation programs during pregnancy. Increasing the awareness of pregnant smokers in regards to pregnancy outcomes and their new social role as mothers, makes pregnancy a "teachable moment," which is important for smoking cessation, as their receptivity towards smoking cessation messages is increased [35].

The majority of smoking cessation interventions converges on providing information on the effects of active smoking on the foetus, emphasizing the advantages of quitting smoking. However, interventions may vary depending on their intensity and approach with common procedures either basic oral counselling [36,37], the provision of self-help manuals booklets or the combination of the above [37,38]. In regards to the intensity of the performed intervention, in most studies an intensive intervention lasting more than 15 minutes has been found to be more effective [39-41] than shorter and less individualized interventions, which are described in some studies as "low intensity interventions" and in others as "usual care" (< 5 minutes) [39,40,42-44]. In a systematic review and meta-analysis performed to evaluate the most effective and shortest counselling intervention for smoking cessation during pregnancy, a more intensive intervention was identified as more effective, if comprised of 15 minutes of cognitive-behavioural orientation accompanied by printed material [45]. The effectiveness of a manual of clinical guidelines for smoking cessation during pregnancy and the role of patient education has been previously evaluated, through which the provision of a self-help manual, a videotape and a brief counselling intervention were found to be more effective in promoting cessation in comparison to the provision of "usual care" (17.3% in the experimental group vs. 8.8% in the control group) [44].

## Hypothesis/aim of the current study

Greece has one of the highest percentages of smoking at a global level, with the percentage of adult female smokers estimated in 2007 at 31.3%, but with geographical differences within parts of the country [45,46]. While smoking prevalence among men over the past few decades has dropped in Greece (from 54% in 1984 to 47% in 1998), the prevalence among women has increased from 19.5% in 1984 to 29% in 1998 [47].What is even more alarming though, is the fact that currently smoking prevalence does not differ by gender in Greece among youth and adolescents, which indicates the need for action to promote smoking prevention and tobacco cessation activities at a national level [48]. With the tobacco epidemic in Greece in mind, the aim of the M-SCOPE study was to create and assess a smoking cessation programme tailor-made for Greek women who smoke during pregnancy that could be applied within the Greek health care system.

Overall, the aim of this clinical trial is to test whether offering pregnant smokers a single high intensity intervention for smoking cessation:

a) Increases the rate of smoking cessation during late pregnancy.

b) Has an effect on adverse birth outcomes attributable to smoking.

c) May promote retaining a smoke free status after childbirth.

d) May reduce the concentrations of circulating biomarkers (cotinine/nicotine) and possibly tobacco related carcinogens, namely 4-(methylnitrosamino)-1-(3-pyridyl)-1-butanol (NNAL) during pregnancy.

## Methods/Design

### Design - Setting

The M-SCOPE study is a randomized controlled trial with a parallel assignment approach that compares the efficacy of a proactive pregnancy-tailored high intensity intervention for smoking cessation, which is the experimental group, with a usual care low intensity intervention, which is the control condition. This research will take place (November 2009-June 2012) in two hospitals situated in the area of Attica, Greece namely through the Peripheral General Maternity Hospital 'Elena Venizelos' (137/04-10-07) and the Maternity Unit of the 'Attikon' University Hospital within Athens (287/30-07-09). Randomisation will be computer generated while the participants will not be aware of their intervention group (single blind).

### Eligibility/Study population

The study population should follow the below criteria in order to be considered eligible to participate, namely: a) currently pregnant, b) currently cigarette smokers of > 5 cigarettes over the past 7 days, and c) > 18 years old. Exclusion criteria include: a) a gestational age > 24 weeks' gestation at the time of entry, b) limited or no telephone access, c) not planning to live at the same address for 1 year, d) unable to read and/or speak Greek fluently, e)current alcohol or substance abusers (defined as strong cravings for alcohol, inability to limit drinking, continued use of alcohol despite the repeated problems) [49], and f) current depression (according to the Greek validated version of the Goldberg's General Health Questionnaire (GHQ)[50,51].

### Data collection points

**Figure 1:**
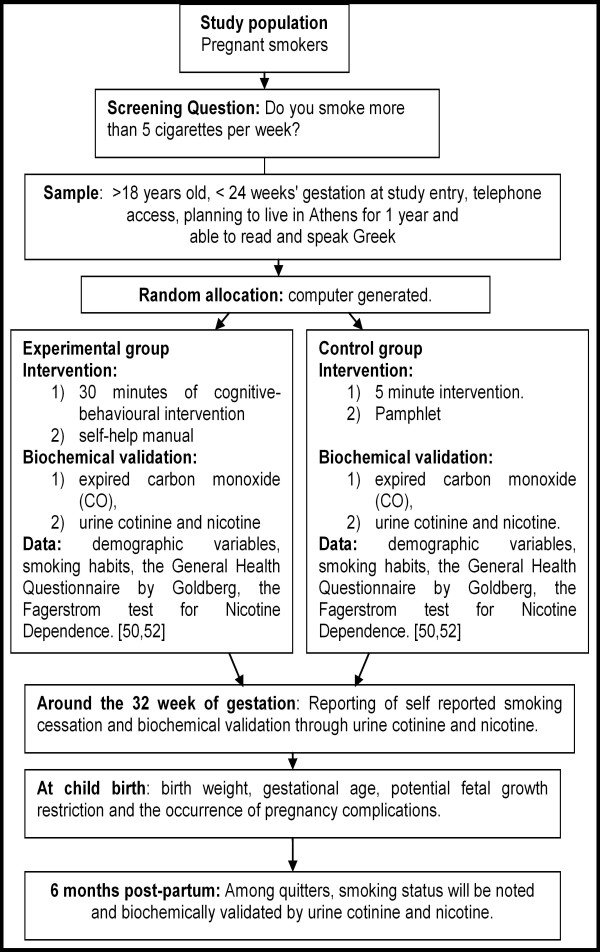
**Study Design Flowchart**.

The different points in this research protocol are described analytically below and illustrated in the study design flowchart depicted in Figure 1.

The first contact (the baseline assessment), will take place before the 24^th ^week of gestation during which prospective participants will be informed about the research aims and procedures, while demographic data and smoking habits are to be recorded.

During the baseline interview the following demographic variables will be recorded: age, weeks of gestation, marital status, ethnicity educational level, current work status. In addition to the above, smoking related data will also be collected (current and past smoking habits, age of smoking initiation, duration of smoking in years, number of cigarettes smoked before pregnancy and currently, partner's smoking habits, prior attempts to quit smoking and duration of these attempts. Furthermore, exposure to secondhand smoke at home, at work and in public places is also to be assessed. Smoking status will be biochemically validated through expired Carbon Monoxide (CO), while urine samples of the participants will be obtained for biomarker analysis. . Additionally during the baseline assessment, the Fagerstrom test for Nicotine dependence [52] and the Greek version of Goldberg's General Health Questionnaire (the 28 question version) are also to be completed [50,51]. During this initial meeting, the intervention will be provided

During the second contact, participants of both groups will receive a follow up phone call before the 32^nd ^week of gestation so as to schedule a second meeting (around the 32^nd ^week of gestation) during which the participants smoking status will be assessed by both self- report and urine sample analysis, which will be collected on that day.

The third contact will take place around childbirth, during which data on birth outcomes and pregnancy complications will be collected.

Finally, six months post-partum, the quitters' post partum relapse will be assessed during a meeting through which smoking status will be reported and again biochemically verified.

### Description of the intervention

Control group participants will receive a face to face low intensity intervention which lasts 5 minutes and will include brief advice and the provision of a leaflet on smoking and pregnancy. This leaflet summarizes the main effects of smoking during pregnancy and gives clear short messages for encouraging smoking cessation by setting up a quit date.

Experimental group participants will receive a higher intensity intervention, which include: 30 minutes of individualized cognitive-behavioural counselling delivered by a trained health care professional and a self-help manual especially tailored for smoking cessation during pregnancy. Counseling will be based at the "5 Αs" (Ask, Advise, Asses, Assist, Arrange). The "5 As" are based on the "four steps" by Glynn and Manley [53] recommended by the US National Cancer Institute The importance of the "5 As" in counselling is corroborated by their recommendation by the US Department of Public Health towards health professionals [33]. Furthermore, in 2010 the American College of Obstetricians and Gynaecologists suggested the "5 Αs" as the intervention of choice for smoking cessation as it is considered short and easy to use [54].

In addition to counselling, a self help manual especially tailored for smoking cessation during pregnancy for Greek women will be provided. According to Lancaster and Stead's systematic review's [55] a self-help manual provides structured materials (printed or audio-visual) that assist the individual in making an attempt to quit and sustaining abstinence without significant assistance from health professionals. For instance, self-help can be provided through printed, video, telephone (recorded messages) or computer-based materials [56]. In most studies a self-help manual is an informative booklet, that is initially presented and explained, and then provided to the pregnant woman to take with her and thus can be consulted and read at home [57,41,42-62]. Such a self-help manual is not limited to providing information about the effects of smoking on the foetus, the potential complications during pregnancy and adverse outcomes in childbirth, but also includes the effects of smoking on women's health or the role of infant second-hand smoke exposure in order to prevent a smoking relapse post-partum [62].

Our self help manual creation was based on the above general rules and principles taking into consideration Greek cultural particularities. In our effort to make a simple but interesting and scientifically oriented self-help manual, photos were included as well and references for further reading were provided. The self-help manual of our protocol was divided into four parts. The first part summarized the key points in regards to the effects of smoking during pregnancy on the foetus, but also of the gain acquired through smoking cessation, the benefits for maternal health and how to prevent relapse, and stay smoke free. Also during the first section a "Questions and Answers" list was included based on common queries brought forward, such as the best time to quit, breast-feeding issues, weight gain, etc. The second part of the self help manual was aimed to prepare the pregnant woman to quit by providing practical solutions for handling cravings and nervousness, the importance of the involvement of the partner and the women's social network. The next step in the manual (third section) was about setting a smoking cessation date. Some general practical suggestions are included as well as some special suggestions for the quit date. More emphasis is given to the strength of her will to remain abstinent. Moreover, on the last page of the self help manual the CDC's document entitled ''within 20 minutes of quitting'' is referenced, which descriptively illustrates what happens to one's body 20 minutes after smoking his/her last cigarette [63].

### Biochemical validation and its justification

Despite the availability of biochemical tests, some studies still accept the trueness of smoking cessation reported by smokers without biochemical validation, even though several studies have refuted the claims of pregnant women through biochemical tests due to high miss reporting [39,64,65]. The importance of biochemical validation of smoking cessation during pregnancy is summarized in a previous systematic literature review, which included 72 RCTs conducted from 1975 to 2008 involving more than 25,000 pregnant smokers, that concluded that studies that do not biochemically validate the smoking status of pregnant women almost certainly have substantial measurement errors and therefore should be considered unreliable [28]. Within the context of our study, smoking status will be both self reported and validated: at the baseline assessment, at around the 32nd week of gestation and approximately 6 months after delivery only for the woman who successfully quit.

Urinary nicotine and cotinine are commonly used to assess exposure to tobacco products. While nicotine has a half-life of only 2-3 hours and can only inform us about recent exposure to tobacco smoke, cotinine (due to its longer half life of 15-19 hours) is a preferred marker of choice [66] Moreover, expired Carbon Monoxide (CO) will be also measured during the encounters. Expired CO will be measured by the Micro Smokerlyzer^® ^CO monitor (Bedfont Scientific Ltd). The same regularly calibrated CO tester will be used for each measurement (Micro CO Tester, Micro Medical Ltd., Rochester, Kent, UK). The expired air CO measurement will be performed according to detailed written guidelines, while the person who will perform the measure will have been trained beforehand. Even though expired CO is a convenient, low-cost measurement, that may provide immediate results for the evaluation of smoking status [67], its short half-life (3-6 hours) can lead to false negatives [68] as it is able to detect only smokers who have smoked within the past several hours and therefore cannot be considered the most reliable biochemical marker for confirming the validity of smoking cessation, but it could be used to motivate pregnant women to quit. [67].

### Description of the laboratory analysis technique

Urine cotinine and nicotine concentrations will be assessed through liquid chromatography mass spectrometry (LC/MS) analysis based on previously applied methods [69,70]. Samples after collection will be allocated into vials and stored at -20 degrees Celsius until the analysis. In order to ensure and increase internal validity the urine cotinine and nicotine analysis will be performed in a separate collaborating laboratory who will be blinded in regards to participant smoking status (smoker/quitter), study group (intervention/control) and sample collection date (pre/post intervention).

#### Reagents

(-)-nicotine (99%) and (-)-cotinine (98%) will be obtained from Sigma-Aldrich (St. Louis, USA). Acetonitrile (LC-MS grade Chromasolv) will be obtained from Fischer Scientific UK Limited (Leicestershire, UK). Ammonium formate (99%), ammonium acetate (98%), methanol (LC-MS grade Chromasolv) and water (LC-MS grade Chromasolv) will be purchased from Fluka Analytical (Steinheim, Germany). Ammonia (25%) will be obtained from Merck (Darmstadt, Germany). Furthermore, solid phase extraction tubes (BOND ELUT-C18, 100 mg, 1 ml) will be purchased from Varian Inc.

#### LC MS analysis

All analyzes will be performed using a liquid chromatograph mass spectrometer (Shimadzu LCMS - 2010 EV) system equipped with an atmospheric pressure chemical ionization (APCI) interface, an autosampler, solvent degasser, binary pump, and a heated/cooled column compartment. The column is a Discovery C18 HPLC Column (25 cm × 4.6 mm, 5 μm, SupelCo, (Bellefonte, USA). Both mass spectrometer and HPLC inlet will be controlled by Shimadzu LCMS solution software (LCMS Solution version 3) which also will be used for data acquisition and processing. The instrument will be tuned and calibrated using autotune procedures recommended by the manufacturer. The detector voltage will be regulated at 1.5 kV and the nebulizing gas flow at 2.5 L/min.

Ten μl (10 μl) from each extracted sample will be entered to the chromatograph column, at temperature 45°C. A gradient of 10 mM ammonium acetate, pH = 5.0, (solvent A) and an acetonitrile (solvent B) are selected for routine use: starting at 10% of solvent B, 90% B (15 min linear ramp), 10% B (5 min). The total mobile phase flow rate will be 0.6 mL/min. The detection will be done in the SIM (selected ion monitoring) positive mode using ion fragments with m/z 163, 204 for nicotine, m/z 177, 218 for cotinine and m/z 238, 279 for ketamine.

### Outcome Measures

The primary outcome assessed will be the participants' smoking status during the 32^nd ^week of gestation. Secondary outcomes include: birth outcomes (such as the infants' birth weight, prematurity of birth, complications during pregnancy) as also smoking relapse among quitters 6 months post partum. Possibly their urinary tobacco specific carcinogens pre/post intervention will be assessed.

### Sample size - Statistical analyses

Sample size was determined based on pilot research during which the rate of smoking cessation was significantly higher in the experimental group (25%) than in the control group (7%). Assuming that in our entire research project might have similar rates of smoking cessation in both groups and for a power of 80% and a significance level of 0.05 this implied a sample size of 128 pregnant smokers.

Statistical analyses will be performed using Statistical Package for the Social Sciences (PASW) version 18. With regard to the process evaluation, descriptive statistics will be used, such as frequencies, means, standard deviations and ranges. General descriptive statistics will be used to describe the participants' demographic data, participants' general and mental health and nicotine dependence, while bivariate and multivariate analyses will also be performed.

### Quality Control

Reliability and validity are considered to be the criteria for assessing the quality of quantitative studies. So as to assure the above, the RCT's randomization list will be kept concealed until the participants' assignment to each group and will be computer generated. After the informed consent form has been signed a study entry number will be assigned to each participant. This number will be on the outside of an envelope, which will allocate accordingly the participant to either the experimental or the control group (single blind allocation). Allocation concealment will prevent the occurrence of selection bias and randomization sequence before and until interventions are provided to the prospective participants [71].

### Ethical Considerations

Prospective participants will have been informed both verbally and in writing about the aims, the methods, the procedures and the measurements performed during this study. They will also be informed about ethical issues such as confidentiality, their right to ask any questions during the study and their right to withdraw at any time. In order to ensure that all participants have received information about this research project and agree to participate, all participants will be asked to sign a written consent form. In all instances research will adhere to the criteria of the World Medical Association Declaration of Helsinki [72].

This research has been approved by the Biomedical Ethics Committee of the Faculty of Medicine of the National and Kapodistrian University of Athens (Protocol approval number: 4568/07-01-08) as also by the Ethics committee of both participating hospitals the Peripheral General Maternity Hospital 'Elena Venizelos' (Protocol approval number: 137/04-10-07) and the Maternity Unit of the 'Attikon' University Hospital within Athens (Protocol approval number: 287/30-07-09). Furthermore the M-SCOPE study trial is registered through clinicaltrials.gov (ClinicalTrials.gov Identifier: NCT01210118).

### Implications

The scope of this study is not only to identify the most effective intervention for smoking cessation during pregnancy in Greece with the use of biochemical measurements, but also to develop such a research protocol for smoking cessation among Greek pregnant women that could be applied within other health care settings in Greece.

The results of this study are important as they could be successfully implemented in clinical practice within Greece, where organized smoking cessation programmes for pregnant smokers are not provided. These programmes could be potentially helpful for both pregnant smokers and their immediate family but also beneficial to the health care system as the cost of such a smoking cessation intervention would likely be minimal in comparison to the treatment of the well known health effects of maternal smoking during pregnancy.

#### Strengths and Limitations

Our study has some important strengths, which are the study design itself (an RCT), and the fact that this is the first RCT to be applied in regards to smoking cessation among pregnant Greek women. Additionally the biochemical verification of smoking status and the collection of biological samples for further analysis also add to the study's strength and research possibilities. On the other hand one possible limitation of this study is that light smoking might be misreported by pregnant smokers, as smoking during pregnancy is not socially accepted and the fact that it is difficult to distinguish at a biomarker level light smoking from heavy exposure to second hand smoke. Another limitation that should be taken into account is the fact that the curriculum of this study may not be generalisable to other countries due of the different cultural background.

## Conclusion

Considering the continuous steady increase in the prevalence of Greek female smokers, such research is needed so as to provide the Greek population with a smoking cessation programme which can be applied within primary health care and maternity care, utilising the "teachable moment" of pregnancy, protecting infants and their mothers from smoking related diseases.

## List of abbreviations used

APCI: Atmospheric Pressure Chemical Ionization; 5 A's: Ask, Advise, Asses, Assist, Arrange; CO: Carbon Monoxide; FRC: Functional Residual Capacity; GHQ: General Health Questionnaire; LC/MS: Liquid Chromatography/Mass Spectrometry; MANOVA: Multivariate Analysis Of Variance; NRT: Nicotine Replacement Therapy; RCT: Randomized Control Trials; SIDS: Sudden Infant Death Syndrome; SIM: Selected Ion Monitoring; TPTEF:TE: Ratio Of Time To Reach Peak Expiratory Flow To Total Expiratory Time; Vmax: Maximal Forced Expiratory Flow.

## Competing interests

The authors declare that they have no competing interests.

## Authors' contributions

Authors ANL and CIV had the main role in manuscript preparation. Authors ANL, ML, PKB, were responsible for research protocol design, while author ANL has the main role in data collection, in which authors GF, CH, CR also contributed. Authors MT and AT contributed to drafting the description of the laboratory analysis technique of this manuscript and will be responsible for the samples' biochemical analyses. Finally authors CIV, GNC and PKB contributed to the project's supervision, manuscript preparation and pilot result interpretation. All authors edited, read and approved the manuscript.

## Funding and Acknowledgements

This research is partially funded by the Smoking and Lung Cancer Research Center of the Hellenic Cancer Society through the Behrakis' Foundation via the "HEART" project (Hellenic Action for Research Against Tobacco).

We would also like to thank the directors and the personnel of both participating hospitals and the colleagues of the Smoking and Lung Cancer Research Center of the Hellenic Cancer Society for their support.

## Pre-publication history

The pre-publication history for this paper can be accessed here:

http://www.biomedcentral.com/1471-2458/11/903/prepub
